# ZMAT3 alleviates cell death in Fanconi anemia via adaptation of sphingolipid metabolism

**DOI:** 10.1126/sciadv.aeb6444

**Published:** 2026-07-31

**Authors:** Maeva Loock, Blandine Lucchini, Emmanuelle Latour, Margot Tissandier, Marie Lhomme, Maharajah Ponnaiah, Lucie Hernandez, Loic Maillard, Jean Soulier, Olivier Bluteau, Camille Lobry, Marie-Laure Arcangeli, Wilfried Le Goff, Dominique Bluteau

**Affiliations:** ^1^ Université Paris-Saclay, Gustave Roussy, CNRS UMR9019, F-94805, Villejuif, France.; ^2^ Université Paris-Saclay, Gustave Roussy, INSERM U1360, F-94805, Villejuif, France.; ^3^ INSERM U1342, Institut de la Leucémie Paris Saint-Louis, Université Paris-Cité, Paris, France.; ^4^ IHU-ICAN, Foundation for Innovation in Cardiometabolism and Nutrition, Paris, France.; ^5^ Université Paris Cité, Institut de Recherche Saint-Louis (IRSL), INSERM UMR1342/CNRS EMR8000, Paris, France.; ^6^ Service de Biochimie Endocrinienne et Oncologique, Assistance publique - Hôpitaux de Paris Sorbonne Université Pitié Salpêtrière, Paris, France.; ^7^ Sorbonne Université, INSERM UMR_S1166, Unité de recherche sur les maladies cardiovasculaires et métaboliques, ICAN, F-75013 Paris, France.; ^8^ Ecole Pratique des Hautes Etudes, PSL University, Paris, France.

## Abstract

Fanconi anemia (FA) is characterized by defective DNA repair and chronic p53 activation, predisposing to acute myeloid leukemia through persistent genomic instability. The molecular adaptations enabling cell survival under chronic stress remain poorly understood. This study investigates the role of ZMAT3, a p53 responsive RNA binding protein overexpressed in FA deficient cells, to elucidate its role in cellular adaptation. Using FA patient fibroblasts and *Fancg* KO mouse models, we launched transcriptomic profiling, lipidomic, and functional analyses. RNA-seq and lipidomic analyses revealed unexpected alterations in sphingolipid metabolism upon ZMAT3 depletion, linked to ceramide accumulation and downregulation of ASAH1 (acid ceramidase). Functionally, ZMAT3 depletion increased DNA damage and enhanced ferroptosis susceptibility, an iron dependent cell death mechanism. Mechanistically, our results showed that ZMAT3 promotes ASAH1 expression, limits ceramide accumulation and protects cells from ferroptosis induced death which could participate to preleukemic clonal evolution and pointing to areas for further exploration in FA-associated malignancies.

## Teaser

ZMAT3 sustains Fanconi anemia cell survival through ceramide regulation, fostering conditions for leukemic evolution.

## INTRODUCTION

*ZMAT3* encodes an RNA-binding protein that is transcriptionally activated by p53 (*1*, *2*) and upregulated in response to various cellular stresses, including genomic instability (*3*) and oxidative stress (*4*). ZMAT3 serves as a positive regulator of p53, thereby establishing a molecular connection between pathways involved in cell senescence, cell cycle, metabolism and insulin resistance in adipose tissue (*5*–*7*). Moreover, integrative analysis of ZMAT3 RNA-binding profile and transcriptomic patterns has unveiled its role on exon inclusion within coding transcripts of diverse functionalities such as splicing regulators and components of numerous cellular processes mediated by the p53 pathway (*8*). Although *ZMAT3* has been identified as a p53-responsive gene, its functional role in cellular physiology remains poorly defined. Emerging evidence indicates that ZMAT3 may act either as a tumor suppressor (*8*, *9*) or as an oncogene (*10*–*12*) depending on cellular context, genetic background and co-occurring molecular alterations. This duality is further highlighted by reports of increased ZMAT3 expression in various malignancies (*13*), including acute myeloid leukemia (AML) (*14*), in which its role remains to be understood.

Fanconi anemia (FA), the most common inherited bone marrow failure syndrome (BMF), offers a model to study early leukemogenesis (*15*). FA arises from biallelic mutations in any of 23 genes essential for interstrand crosslink (ICL) repair (*16*) and genome stability (*16*). FA is characterized by early hematopoietic stem progenitor cell (HSPC) loss, cytopenia and chromosomal instability, creating an environment conducive to clonal evolution and progression to myelodysplastic syndrome (MDS) and AML. FA HSPCs experience chronic stress with persistent p53 activation and apoptosis (*17*) driven by defective DNA repair, hypersensitivity to aldehyde (*18*, *19*), inflammatory cytokines (*20*, *21*), oxidative stress (*22*) and a hyperactive TGF-β pathway (*23*). This persistent genomic instability is thought to facilitate the accumulation of additional somatic mutations, progressively driving hematopoietic cells toward malignant transformation. We hypothesized that *TP53* alterations might underlie FA related leukemogenesis. However, genomic analyses across disease stages revealed only rare *TP53* mutations but frequent amplifications of *MDM2/MDM4*, key p53 inhibitors (*24*, *25*). Strikingly, 1q gain, harboring *MDM4*, emerged as the most frequent early oncogenic event (∼50% of MDS/AML cases). Cross-cancer analyses confirmed the dependence on MDM4 in *TP53* wild-type contexts, highlighting its importance as an alternative to p53 suppression during early tumor evolution (*26*). FA represents a unique constitutional model for studying chronic p53 activation and its downstream effects. This rare autosomal recessive genetic disorder, characterized by defects in ICL repair pathways, generates persistent genomic instability leading to chronic activation of p53 dependent signaling (*27*). These findings led us to investigate whether early dysregulation of p53 targets in FA cells might promote cell survival in a context of genomic instability, while potentially contributing to the creation of a cellular environment more prone to transformation.

To address how cells with DNA repair deficiencies achieve stress adaptation and undergo clonal selection, we implemented an integrative experimental approach encompassing cellular, molecular, and lipidomic methodologies. These combined investigations revealed a pivotal role of ZMAT3 in regulating cell fate plasticity through sphingolipids reprogramming, disclosing it as a potential determinant of clonal selection in FA deficient cells. These results highlight how lesser known p53 effectors can paradoxically support stressed cell survival while creating conditions that promote secondary oncogenic events in cancers retaining functional *TP53*.

## RESULTS

### ZMAT3 is upregulated in FA-deficient cells, and its depletion leads to alterations in both the transcriptomic and lipidomic profiles

**Fig. 1.**
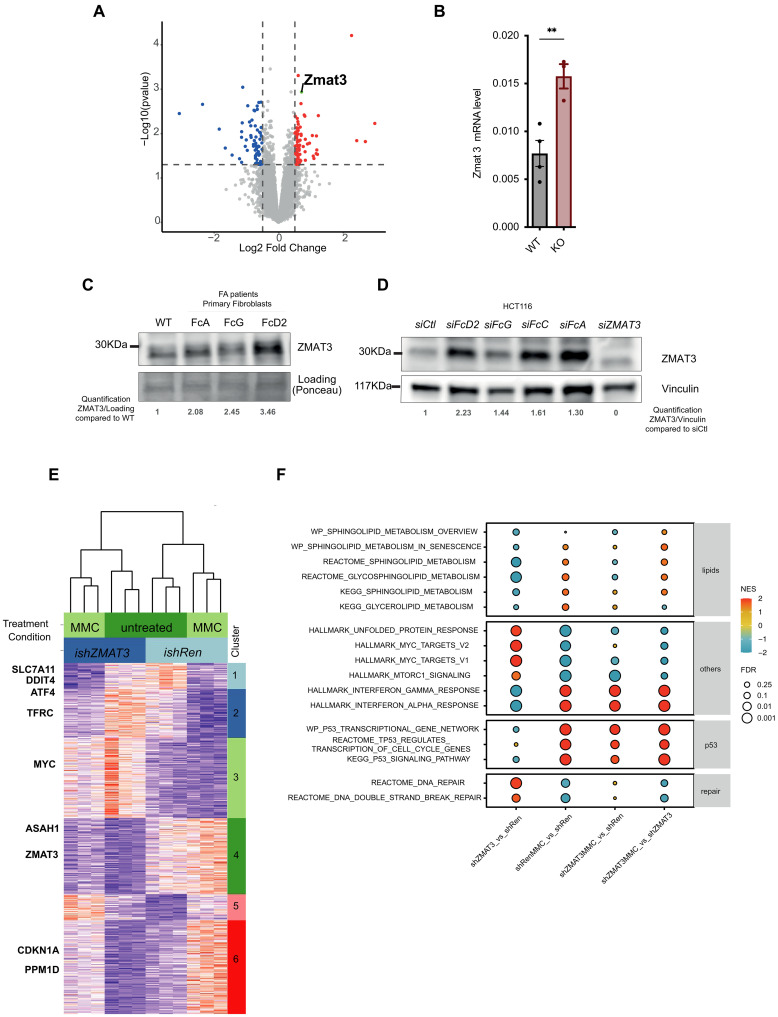
ZMAT3 is upregulated in FA-deficient cells, and its depletion leads to alterations in both the transcriptomic and lipidomic profiles. (**A**) Volcano plot from RNAseq experiments comparing *Fancg* KO LSK cells to WT LSK cells mRNA expression (from 6 KO and 6 WT mice). (**B**) *Zmat3* mRNA expression level normalizes to *Hprt* into *WT* (*n* = 4), *WT_pIC* (*n* = 3),* KO *(*n = *4)* and KO_pIC *(*n = *4) mice LSK cells. **P < 0.01. (**C**) Western blot analysis of ZMAT3 in three primary fibroblasts cell line from FA patients, each carrying distinct mutations in the FA pathway: in *FANCA (FcA)*, *FANCG (FcG)* and *FANCD2 (FcD2)*. WT corresponding to primary fibroblast cell line without FA genes mutation. (**D**) Western blot analysis of ZMAT3 in HCT116 cells 48 hours post siRNA transfection against different Fanconi genes compared to control (*n* = 3; one representative experiment is shown). (**E**) Heatmap with hierarchical clustering of RNA-Seq data showing significant differential gene expression between PD20ish*ZMAT3* and control PD20ish*Ren* cells, 2 days post-treatment with doxycycline (DOX, 0.5 μg/ml) in the presence or absence of mitomycin C (MMC, 30 nM). (**F**) GSEA analysis.

We and others provide evidence that early adaptive changes occur in FA cells within the p53 pathway leading us to hypothesize that adaptation to p53 signaling may prime cells for oncogenic progression. To address this, we used an FA HSPC model to identify dysregulated p53 target genes, together with FANCD2-mutant fibroblasts to investigate their functional roles. First, we investigated the effects of FA pathway disruption in HSPCs from *Fancg^−/−^* (KO) mice. Transcriptomic profiling revealed, in accordance with previous studies, p53 pathway hyperactivation and enrichment of apoptotic, metabolic and MYC-associated pathways (*17*, *28*). Among a limited set of deregulated genes belonging to p53 pathway, *Zmat3* was markedly overexpressed (Fig. 1, A and B) (*29*, *30*). This upregulation was also observed after depletion of four different FA genes in HCT116 cells and in primary fibroblasts cell line derived from three patients carrying distinct mutations in the FA pathway compared to control (siRNA control in HCT116 and primary fibroblast cell line without mutation in FA genes pathway respectively) (Fig. 1, C and D), suggesting that ZMAT3 activation is a common response to FA associated genomic stress.

### ZMAT3 depletion leads to ASAH1 depletion triggering ceramide accumulation in FA-deficient cells

**Fig. 2.**
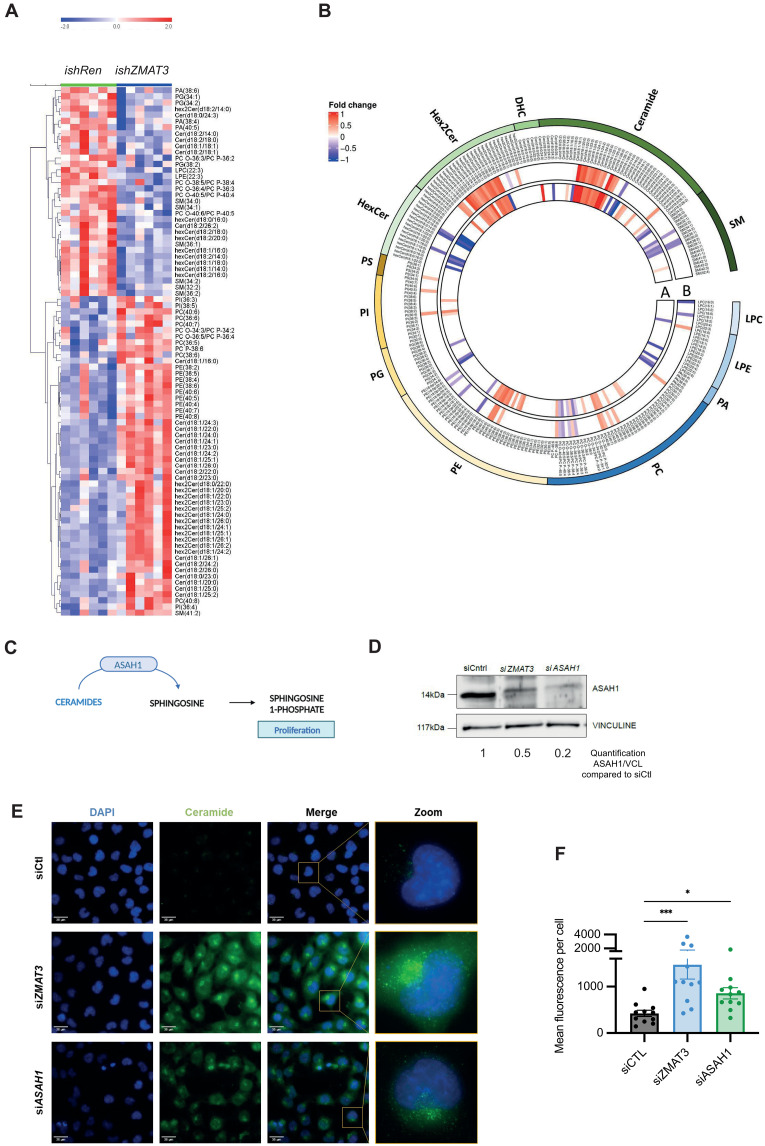
ZMAT3 depletion leads to ASAH1 depletion triggering ceramide accumulation in FA-deficient cells. (**A**) Lipidomic heatmap comparing PD20ish*ZMAT3* and control PD20ish*Ren* cells (*n* = 6). Lipids were quantified by LC-ESI/MS/MS using a Prominence UFLC and QTrap 4000 system. Phospholipid and sphingolipid analysis were analyzed onto a Kinetex HILIC column (2.6 μm, 2.1 × 150 mm). Statistical analysis and clustering were performed with MetaboAnalyst 5.0. (**B**) Circos plot representation of lipidomic experiments highlighting lipid species enrichment comparing PD20 cells *ZMAT3* depleted to control (*n* = 6 per group). A: *ishZMAT3* vs i*shRen* (untreated); B: *ishZMAT3* vs i*shRen* upon MMC treatment. (**C**) Schematic representation. (**D**) Western blot analysis of ASAH1 protein in PD20 depleted by RNA interference against ZMAT3 and *ASAH1* compared to control (*n* = 3, one representative experiment is shown). (**E**) Immunofluorescence using ceramides antibody in PD20 depleted by RNA interference against *ZMAT3* and *ASAH1* compared to control. Shown is one representative experiment (*n* = 3). (**F**) Quantitative analysis of the mean fluorescence intensity signaling per cell in PD20 cells post-48-hour siRNA transfection (*n* = 3 per group, 3–4 images/per group/experiment with at least 30 cells per image). Errors bars shown mean+/− SEM. *P* values from ANOVA kruskal-wallis test are shown. *P < 0.05; ***P < 0.001.

To investigate ZMAT3 functional role in the FA deficient cells, we used a human fibroblast cell line derived from an FA patient [PD20 cells, (*31*)] as a surrogate model. Indeed, studying FA deficient HSCs is a significant challenge since, beyond their rarity and obvious ethical reason, they are difficult to isolate and to maintain due to their high mortality rate. We generated stable PD20 lines expressing an inducible shRNA targeting *ZMAT3* (ish*ZMAT3*) or a control (ish*Ren*) via lentiviral transduction (fig. S1). RNA-seq analysis was then performed to identify dysregulated genes and pathways associated with *ZMAT3* depletion. Cells were analyzed under baseline conditions but also following mitomycin-C (MMC) treatment (48 hours, 30 nM), an inducer of ICLs, to mimic the genomic instability observed in FA. We established the transcriptomic profiles and identified differentially expressed genes between *ZMAT3* depleted and control cells with or without MMC treatment corresponding to six clusters (Fig. 1E and table S1). As expected, in MMC treated PD20 control cells, gene set enrichment analysis (GSEA) analysis showed an enrichment of genes related to pathways such as p53 Pathway (which includes ZMAT3, CDKN1A, PPM1D), Interferon alpha and gamma response, TNF and inflammatory pathways (Fig. 1F). In untreated conditions, ZMAT3 depletion in PD20 cells led to the dysregulation of gene expression affecting pathways related to DNA repair, mTORC1 signaling and MYC targets (fig. S2). Unexpectedly, GSEA also revealed significant perturbations in several lipid metabolism pathways, both at untreated and following MMC treatment (Fig. 1E). Therefore, we performed in-depth lipidomic profiling to investigate the metabolic consequences of ZMAT3 loss. Lipidomic analysis confirmed alterations in the lipid landscape upon *ZMAT3* silencing. Specifically, we observed marked changes in glycerophospholipids, particularly phosphatidylethanolamines (PE), and sphingolipids alongside a notable accumulation of ceramides (Fig. 2, A and B, table S2, and fig. S3). The accumulation of ceramides, key regulators of cell fate decisions such as apoptosis, suggested a potential functional link between ZMAT3 and sphingolipid homeostasis. In light with this lipidomic results we reanalyzed the RNA-seq data focusing on genes involved in sphingolipid metabolism. We evidenced a significant downregulation of ASAH1 in *ZMAT3* depleted cells (Fig. 1E). ASAH1 encodes acid ceramidase, responsible for hydrolyzing ceramides with C6–C16 acyl chains into sphingosine (Fig. 2C). This sphingosine is then converted into sphingosine-1-phosphate (S1P) by sphingosine kinases SK1/2, which play crucial roles in promoting cell survival and proliferation (*32*). We validated the downregulation of ASAH1 at protein levels in the PD20 cells depleted for *ZMAT3* using siRNA strategy (Fig. 2D). In silico analysis using the AREsite2 database revealed multiple AU-rich regions in the ASAH1 3′UTR, which may represent potential ZMAT3 binding sites involved in post-transcriptional regulation. We thus hypothesized that the reduction in ASAH1 acid ceramidase activity would contribute to ceramide accumulation in *ZMAT3* depleted PD20 cells. Consistent with our hypothesis, knockdown of either *ZMAT3* or *ASAH1* in PD20 cells led to an increase in ceramide levels (Fig. 2, E and F). All together, these findings suggest that ZMAT3 depletion alters molecular pathways involved in cellular stress responses, DNA repair and metabolism, which are particularly relevant in the context of FA. Furthermore, we uncover a new role for ZMAT3 in regulating sphingolipid metabolism in cellular model with defects in the FA DNA repair pathway.

### ZMAT3 and ASAH1 depletion enhances DNA damages and ferroptosis-induced cell death in FA-deficient cells post genotoxic exposure

**Fig. 3.**
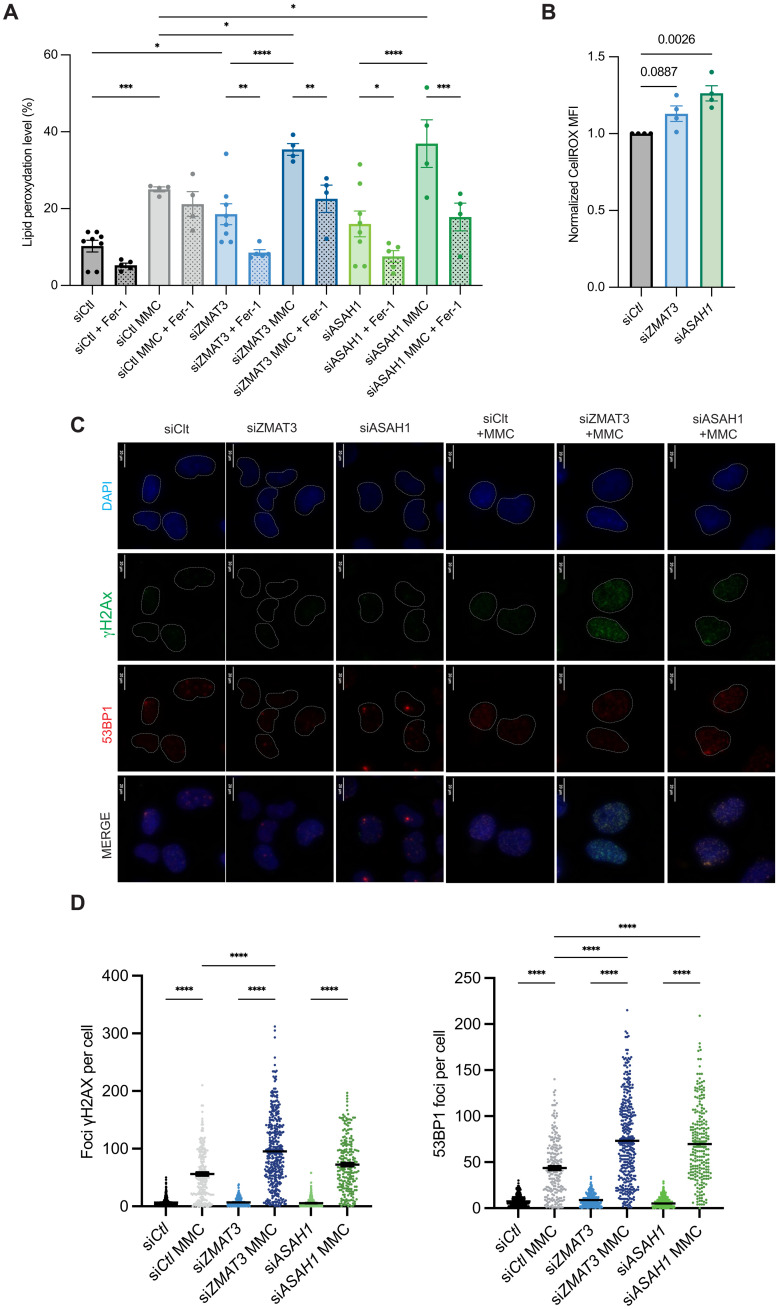
ZMAT3 and ASAH1 depletion enhances DNA damages and ferroptosis-induced cell death in FA-deficient cells post genotoxic exposure. (**A**) Quantification of cellular lipid peroxidation in PD20 cells 24 hours post-siRNA transfection, with or without Ferrostatin-1 (Fer-1, 500 nM) treatment and a 2-hour MMC pulse (300 ng/ml), followed by a 24-hour recovery period. Lipid peroxidation levels corresponding to green positive cell percentage were measured using the C11-BODIPY dye. Errors bars shown mean+/− SEM. *P* values from ordinary one ways ANOVA are shown (siRNA alone *n* = 8, including drugs *n* = 5). (**B**) Quantification of mean fluorescence intensity (MFI) of cellular ROS level in sorted PD20 cells post 48 hours siRNA transfection, measured by the CellROX dye. Errors bars shown mean+/− SEM. *P* values from ordinary one ways ANOVA are shown (*n* = 4). (**C**) Fluorescence microscopy images showing the presence of 53BP1 and γH2AX foci in PD20 cells post-48-hour siRNA transfection, with and without a 2-hour MMC pulse (300 ng/ml), followed by a 24-hour recovery period. (**D**) Quantification analysis of γH2AX foci and of 53BP1 per cell. Errors bars shown mean+/− SEM. *P* values from ANOVA kruskal-wallis are shown. *P < 0.05, **P < 0.01, ***P < 0.001, and ****P < 0.0001. Shown is one representative experiment (*n* = 3).

We next investigated the impact of ZMAT3 or ASAH1 depletion on cellular outcomes. We performed phenotypic analyses of PD20 cells with siRNA mediated knockdown of *ZMAT3* or ASAH1 under untreated conditions and following MMC induced genotoxic stress to assess apoptosis, proliferation and senescence pathways in which ZMAT3 has been previously implicated. In untreated condition, post ZMAT3 or ASAH1 depletion, CellTrace analysis revealed a modest but significant decrease in proliferation, reflected by higher normalized MFI values (fig. S4A), without significant changes in senescence, necrosis or apoptosis rates (fig. S4, B and D). Moreover, in line with prior findings, we observed an increase in PD20 cells senescence and apoptosis following exposure to MMC (fig. S4C). Interestingly, beyond apoptosis, FA cells exhibit increased susceptibility to ferroptosis (*33*, *34*). Ferroptosis is characterized by lipid peroxidation and reactive oxygen species (ROS) accumulation and can be triggered in response to DNA damage (*35*, *36*). To further investigate ZMAT3 potential role in ferroptosis, we measured lipid peroxidation and ROS levels following *ZMAT3* depletion using the lipid-specific dye BODIPY C11 and CELLROX which respectively quantify lipid peroxidation and cellular ROS accumulation. We observed a significant increase in lipid peroxidation upon *ZMAT3* depletion (Fig. 3A), along with a trend toward elevated ROS levels (Fig. 3B). Interestingly, depletion of ASAH1, in the same conditions, phenocopied observations obtained in ZMAT3-depletion context (Fig. 3, A and B). Additionally, escalating doses of Erastin or RSL3, well-established ferroptosis inducers, resulted in reduced cell viability, an effect exacerbated by *ZMAT3* depletion as well as ASAH1 depletion (fig. S4E). Accordingly with these results, using RNA-seq data, we identified ferroptosis as an enriched KEGG pathway in Cluster 1, with a deregulation of key ferroptosis protective genes in PD20ish*ZMAT3* cells in both conditions (untreated or MMC treatment), compared to PD20ish*Ren* cells (Fig. 1E and fig. S2). Furthermore, focusing GSEA analysis using two dedicate ferroptosis pathway gene lists, we confirmed this observation (fig. S2B). As ZMAT3 was shown to stabilize and modulate splicing of *CD44* mRNA, a potential effector of ferroptosis (*27*), we performed also quantitative RT-PCR and showed that ZMAT3 depletion in PD20 cells led to a moderate decrease of total mRNA CD44 transcripts and also its short isoform (fig. S2C).

Subsequently, we assessed lipid peroxidation as a marker of ferroptosis following MMC treatment, with or without ferrostatin-1, a ferroptosis inhibitor, using BODIPY-C11 staining (Fig. 3A). As expected, MMC exposure increased lipid peroxidation in control PD20 cells and treatment with ferrostatin attenuated lipid peroxidation across all conditions, with a significant protective effect observed specifically post ZMAT3 or ASAH1depletion (Fig. 3A).

To investigate if DNA damages act as a trigger of ferroptosis in this context, we evaluated the impact of *ZMAT3* and ASAH1 depletion on DNA damage response. Thus, siRNA mediated knockdown of *ZMAT3* was performed in PD20 cells under untreated and following MMC induced genotoxic stress. DNA damages were evaluated by immunofluorescence staining of γH2AX and 53BP1, with quantification of nuclear foci in untreated (control) and MMC-treated cells. In untreated condition, ZMAT3 or ASAH1 depletion did not significantly affect DNA damage levels, however following MMC treatment, the number of foci was markedly increased in all siRNA conditions showing a significant accumulation of damages post ZMAT3 or ASAH1 depletion (Fig. 3D). These results support the role of ZMAT3 and ASAH1 in limiting DNA damage accumulation under genotoxic stress. Altogether, we identified a novel protective role of ZMAT3 and ASAH1 in limiting DNA damage and modulating ferroptosis, highlighting their importance in maintaining genomic stability in FA deficient cells.

### ZMAT3 depletion triggers ceramide accumulation in *Fancg* KO hematopoietic cells

**Fig. 4.**
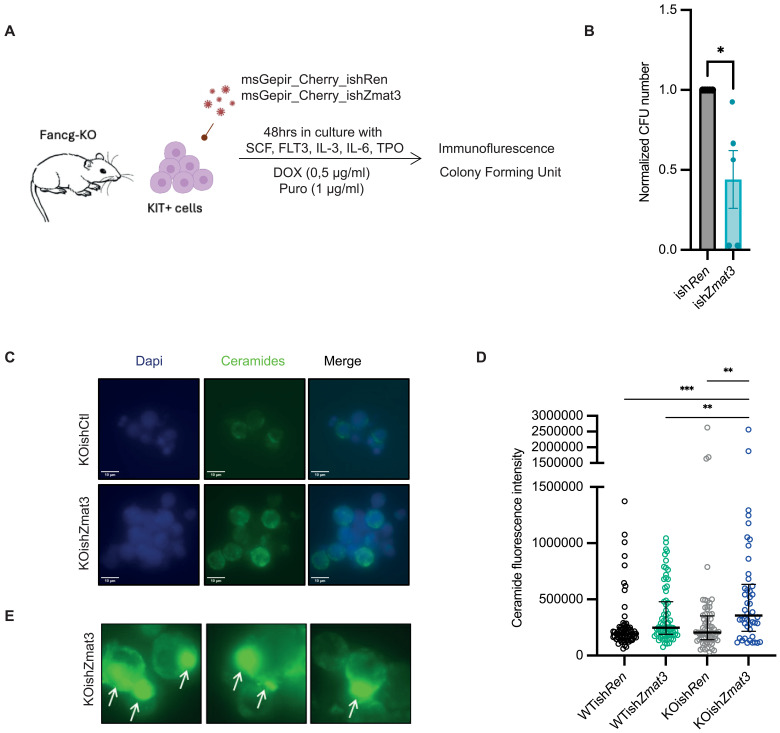
ZMAT3 depletion triggers ceramide accumulation in KO HSPCs. (**A**) Experimental strategy to CFU analysis. (Author created illustrations using PowerPoint.) (**B**) CFU number from *Fancg* KO Kit^+^mcherry^+^ sorted cells post sh*Ren* or sh*Zmat3* transduction (*n* = 5). *P* values from ordinary one-way ANOVA are shown. (**C**) Ceramide distribution in KO hematopoietic cells, either ishRen (control) or ish*Zmat3*-transduced, visualized through immunofluorescence microscopy. (**D**) Quantitative analysis of the fluorescence intensity signaling ceramide presence in mCherry^+^ WT or KO hematopoietic cells, comparing ish*Ren* and ish*Zmat3* conditions. Shown is one representative experiment. *P* values from ordinary one-way ANOVA are shown. (*n* = 2). (**E**) Detailed immunofluorescence microscopy highlighting ceramide accumulation in ish*Zmat3*-transduced KO hematopoietic cells, with specific focus on punctate ceramide signal aggregation indicated by arrows. *P < 0.05; **P < 0.01; ***P < 0.001.

To further explore the relevance of our findings, we examined whether the role of ZMAT3 identified in the *FANCD2* mutant fibroblast model could operate in FA pathogenic context of HSPCs in which genomic instability drives BMF. We thus isolated Lin^−^ KIT^+^ cells, containing hematopoietic progenitors and HSC) from *Fancg* KO mice bone marrow (BM) and transduced them with inducible shRNA against *Zmat3* or a control (ish*Zmat3* and ish*Ren*, respectively). Thus, we performed a colony forming unit assay (CFU) (Fig. 4A) and we showed that *Zmat3* depletion results in fewer colonies than in KO control condition (*ishRen*) (Fig. 4B) confirming our observation in PD20 cells. We also evaluated the effect of Zmat3 silencing on CFU formation in a non-FA background using Lin-Kit^+^ cells and did not observe any difference in CFU numbers. These results suggest that the impairment of CFU formation upon Zmat3 depletion is specific to the FA context. Subsequent ceramide immunofluorescence analysis confirmed that *Zmat3* depletion in these KO hematopoietic cells lead to ceramide accumulation, as evidenced by an intensified signal compared to WT (Fig. 4, C and D). Notably, cells with *Zmat3* knockdown exhibited a 10% incidence of ceramide signal clustering (Fig. 4E).

Collectively, these findings uncover the pivotal role of ZMAT3 in regulating sphingolipid metabolism and preventing ceramide build-up preserving the functional integrity of hematopoietic FA deficient cells.

## DISCUSSION

In this study, we identified ZMAT3, a p53-responsive RNA-binding protein, as a key mediator of cellular adaptation in FA deficient cells. Using a surrogate model of FA patient HSCs we identified a previously unrecognized role for ZMAT3 in the cellular response to p53 activation. We demonstrate that ZMAT3 regulates sphingolipid metabolism by promoting ASAH1 (acid ceramidase) expression, thereby preventing pathological ceramide accumulation. We also establish a previously unrecognized function of ZMAT3 in protecting cells from ferroptosis under conditions of genomic instability. ZMAT3 depletion leads to increased sensitivity to ferroptosis, accompanied by a marked accumulation of phosphatidyl-ethanolamines (PEs) and ceramides. This lipidomic profile closely resembles that observed in cells treated with ferroptosis inducers, characterized by an increase in PE enriched with polyunsaturated fatty acids (PUFAs), which are substrates for peroxidation (*37*) and ceramide (*38*). We identified that ZMAT3 promotes ASAH1 protein synthesis, thereby limiting the abnormal accumulation of ceramides. A previous study in melanoma cell lines reported that loss of ASAH1 leads to ceramide accumulation and elevated peroxisome derived ROS (*39*). In our context, ZMAT3, by promoting ASAH1 expression, may serve as a mechanism to prevent ceramide accumulation, thereby mitigating ferroptosis-associated cell death. Finally, we identified an accumulation of ceramides in FA deficient hematopoietic Lin- Kit+ cells when *Zmat3* is depleted.

The mechanisms involved in this response are transposable to *Fancg*^−/−^ HSPCs. Our results demonstrate that the presence of *Zmat3* in *Fancg*^−/−^ HSPCs sustains colony formation. This observation is particularly relevant considering previous reports indicating that ZMAT3 is upregulated under conditions associated with HSPC decline (*40*, *41*), suggesting that *ZMAT3* overexpression may act as a compensatory mechanism to preserve hematopoietic progenitor cell function under cellular stress. Metabolic reprogramming plays a pivotal role in the self-renewal and differentiation of HSCs, especially during the shift from quiescence to activation, through a complex rearrangement of metabolic pathways. This allows HSCs to modulate their energy consumption in response to activation signals, maintaining a balance between resting for long term self-renewal and activating for differentiation. Lipids, notably sphingolipids, have emerged as key players in HSC metabolic reprogramming and differentiation (*42*). The impact of ceramide/dihydroceramide balance on activating stress responses in HSPCs (*42*) suggests a role for ZMAT3 in HSPC differentiation and self-renewal, through its impact on sphingolipid metabolism. Recent literature has revealed dual and context dependent roles for ZMAT3, acting either as a tumor suppressor (*8*, *9*) or as an oncogene (*10*–*12*), influenced by genetic background and cellular conditions. Our study aligns with and extends these findings by demonstrating a protective role of ZMAT3 in the deregulated FA pathway context.

One mechanism by which ZMAT3 supports cell survival involves increased expression of ASAH1, a ceramidase whose expression and enzymatic activity have been shown to be elevated in AML primary cells (*43*). Targeting ASAH1 has emerged as a promising therapeutic strategy in oncology. The specific ASAH1 inhibitor LCL-805 has shown efficacy in AML by blocking AKT signaling and promoting a form of iron dependent cell death (*44*). Furthermore, pharmacological inhibition of ASAH1 using LCL521 or Carmofur in colorectal cancer cell lines has been reported to enhance radiosensitivity to x-rays (*45*). Thus, we reveal a new connection between ZMAT3 and ASAH1, supporting further investigation in the context of AML. This protective role appears to support cell survival despite persistent DNA damage. Such survival of genomically unstable cells may facilitate the accumulation of early oncogenic changes, thereby contributing to preleukemic clonal evolution. These results highlight how lesser known p53 effectors can paradoxically support stressed cell survival while creating conditions that may promote secondary oncogenic events in cancers retaining functional *TP53*. We also highlight ZMAT3 as a potential mediator linking lipid metabolism, cell death pathways, and the early stages of leukemogenesis, opening new avenues for understanding the mechanisms driving malignant transformation (*24*, *25*).

Given that ZMAT3 is a transcriptional target of p53 and its expression is upregulated in a p53 dependent manner, our findings offer valuable insights into the potential implications of ZMAT3 in cancers presenting wild-type *TP53*, where DNA repair pathways are compromised. This is particularly relevant considering that numerous cancers retain wild-type *TP53*, highlighting the necessity to better understand the functional roles of specific p53 target genes in tumor development and cellular adaptation to chronic stress.

## MATERIALS AND METHODS

### Animal model and facility

Fancg^−/−^ mice (KO) were a kind gift of F. Arwert (*46*). All mice were housed and handled in the pathogen-free animal facility Département d’Expérimentation Animale in accordance with Institutional Animal Care and Use Committee-approved protocols (IRSL, Saint-Louis Hospital). Authorizations for animal experimentation was obtained with the no. 2014-IUH013 and APAFIS#19958-2019032615074625v2, in accordance with French laws.

### Cell lines and culture conditions

SV40-transformed fibroblasts derived from individual diagnosed with Fanconi Anemia, specifically the FA group D2 (PD20 cells), were acquired from the Fanconi Anemia Cell Repository housed at Oregon Health and Science University (*31*). PD20 cells were cultured in alpha-minimum essential medium (MEM, Gibco), enriched with 10% FBS and fortified with 1% Pen/Strep. Mycoplasma contamination was excluded using the MycoAlert Detection Kit (Lonza).

### Lentiviral particle production

Transfection of HEK293T cells was carried out to facilitate the production of lentiviral particles. Cells were initially cultured in Dulbecco’s Modified Eagle Medium (DMEM, Gibco) supplemented with 10% fetal bovine serum (FBS), 1% Penicillin/Streptomycin (Pen/Strep), and 1% GlutaMAX, ensuring optimal growth conditions. Upon reaching 70–80% confluence, cells were transfected with the plasmid of interest along with helper plasmids pCMV and pMD2G, essential for viral assembly and packaging. Transfection was conducted using JetPrime Reagent and Buffer (PolyPlus), following the manufacturer’s protocol. Following an overnight incubation, the culture medium was replaced with DMEM supplemented with 5% FBS and 1% Pen/Strep. Viral supernatants were collected at two time points, 24 and 48 hours post-medium change. Each collection involved a centrifugation step at 3000 rpm for 10 minutes at 4°C to remove cellular debris, followed by ultracentrifugation at 22,000 rpm for 70 minutes at 4°C, concentrating the viral particles. The resulting viral pellet was carefully resuspended in phosphate-buffered saline (PBS) and left overnight at 4°C to ensure complete dissolution. This preparation was then aliquoted and stored at −80°C for experimental use.

### Isolation and viral transduction of HSPCs

HSPCs were isolated from the bone marrow (BM) of *Fancg* KO mice, obtained through collaboration with Pr Jean Soulier’s team at Saint-Louis Hospital. The isolation procedure involved flushing the hind limb bone to harvest bone marrow cells, followed by red blood cell lysis using ACK Lysing Buffer (Thermofisher). Lineage-positive (Lin^+^) cells were depleted through immuno-magnetic separation employing the Lineage Cell Depletion mouse Kit (Miltenyi Biotec), after which Kit^+^ cell enrichment was conducted using the CD117 MicroBeads mouse Kit (Miltenyi Biotec). Following isolation, HSPCs underwent viral transduction in BIT 9500 Serum Substitute (StemCellTM) enriched with 2% Pen/Strep. The viral vectors carrying plasmids msGepir-Cherry-ish*Zmat3* or msGepir-Cherry-ishRenilla (control) were introduced to the cells with the transduction efficiency determined at a ratio of 100,000 cells to 5 μl of virus, within 96-well plates. A 24-hour post-transduction incubation allowed for subsequent treatment with a cytokine cocktail (100 ng/ml SCF, 100 ng/ml FLT3, 20 ng/ml TPO, 20 ng/ml IL-3, and 10 ng/ml IL-6). To select for successfully transduced cells, 1 μg/ml puromycin was applied, and 0.5 μg/ml doxycycline was used to induce plasmid expression over a 48-hour period.

### CFU-C assays

After a 48-hour period of puromycin selection and plasmid induction with doxycycline, HSPCs were subjected to cell sorting using an AriaFusion BD system, specifically targeting Cherry-positive cells. Dead cells were excluded through application of SYTOX Blue Dead Cell Staining (Invitrogen) at a dilution of 1:1000. The sorted Cherry-positive HSPCs were then cultured in duplicate in methylcellulose-based medium specifically designed for mouse cells MethoCult GF M3434 (StemCellTM), which was further supplemented with 2% Penicillin/Streptomycin and doxycycline at a concentration of 0.5 μg/ml. The cultures were maintained at 37°C in a 5% CO_2_ atmosphere, with doxycycline supplementation renewed every 48 hours. Colony formation was assessed after 7 days of incubation.

### Ceramides detection in HSPCs by immunofluorescence

For assessment of ceramide in HSPC, following a 48-hour period of selection with puromycin and induction of plasmid expression using doxycycline, 20,000 cells from each condition were isolated and fixed with 4% paraformaldehyde for 15 minutes at room temperature. Subsequently, the cells were washed in PBS and adhered to Poly-Prep slides (P0425–72EA, Sigma) overnight at 37°C, followed by permeabilization with 0.5% Triton X-100 in PBS for 10 minutes at room temperature and another PBS wash. Initial incubation was performed for 1 hour in a saturation buffer containing 3% BSA in TBS 1X with 0.1% Tween, after which cells were stained with anti-ceramide antibody (MID 15B4, Enzo Life Sciences) for 1.5 hours at room temperature. After three washes, cells were incubated with secondary antibodies, goat-anti-mouse-IgM (SA5–10150, ThermoFisher), for 1 hour, followed by three additional washes, and finally stained with DAPI in mounting medium. Images were acquired on an Axio Imager Z1 microscope using the Axio Vision software (Zeiss).

### Plasmid transduction

Cells were plated at 2.0 × 10^5^ per well in six-well plates 24 hours before lentiviral infection. For transduction, 10 μl of lentiviral particles were added to each well and incubated for 24 hours to facilitate viral entry and integration. Post-incubation, cells were washed and incubated for an additional 48 hours to allow expression of selection marker. Subsequently, cells underwent selection with 2.5 μg/ml puromycin to isolate successfully transduced cells.

### siRNA transfection and treatment

For transient depletion of *ZMAT3* and *ASAH1* expression in PD20 cells, siRNAs obtained from Dharmacon (On-TARGET plus SMART pool) were employed. As a control, unspecific siRNAs (siCntrl) were used. Cells were transfected with 30 nM of siRNAs using JetPrime Reagent and Buffer (PolyPlus) according to the manufacturer’s instructions.

Within designated experimental conditions, cells underwent a pulse treatment with Mitomycin C (MMC) at a concentration of 300 ng/ml for 2 hours, initiated 24 hours following transfection. After this pulse exposure, cells were thoroughly washed and provided with fresh culture media. This step was succeeded by a continued incubation period of 24 hours at 37°C in an atmosphere containing 5% CO_2_ to allow for cellular recovery and response evaluation. When specify, 500 nM Ferrostatin-1 (Fer-1) was added in media 8 hours after transfection to inhibit ferroptosis. Post-MMC pulse, Fer-1 was introduced in fresh media.

### Apoptosis, necrosis, senescence, and ferroptosis assay

Cells were plated at 1.0 × 10^5^ per well in six-well plates 24 hours prior to siRNA transfection. Cells were treated as indicated, then to assess apoptosis and necrosis and senescence. Cells were harvested and stained with annexin V-AlexaFluor 647 (BioLegend) at a 1:100 dilution in 1X Apoptosis Binding Buffer (Invitrogen) for 15 minutes at room temperature, followed by a wash and subsequent staining with SYTOX Blue Dead Cell Staining (Invitrogen) at a 1:1000 dilution in 1X Apoptosis Binding Buffer (Invitrogen). For ferroptosis detection, cells were similarly collected and then incubated in PBS containing BODIPY 581/591 C11 (Thermofisher, D3861) at a concentration of 750 nM for 20 minutes, followed by washing. For senescence detection, we used CellEvent Senescence Green Detection Kit (Thermofisher, C10850) according to the manufacturer’s instructions. Flow cytometry analysis was conducted using a CytoFLEX flow cytometer system to evaluate cell death, lipid peroxydation or senescence markers.

### Proliferation assay

Cells were plated at 1.0 × 10^5^ per well in six-well plates 24 hours prior to siRNA transfection. The assessment of cell proliferation was facilitated using the CellTrace Blue Cell Proliferation Kit (Thermofisher, C34568), according to the manufacturer’s instructions. Following a 72-hour incubation period, proliferation rates were quantified via flow cytometry analysis, employing a Fortessa flow cytometer instrument.

### Cell survival assay

Cells were plated at 1.0 × 10^5^ per well in six-well plates 24 hours prior to siRNA transfection. Following transfection, a period of 6 hours was allowed before cells were re-plated into 96-well plates at a concentration of 4000 cells per well. After an overnight incubation period, cells were exposed to an escalating series of erastin concentrations for a duration of 48 hours to evaluate drug sensitivity. Subsequently, cell viability was determined using the CyQUANT MTT Cell Viability Assay (Thermofisher, V13154), according to the manufacturer’s instructions.

### Ceramides and DNA damage detection by immunofluorescence in cell lines

Cells were plated at 1.5 × 10^5^ per well in six-well plates 24 hours prior to siRNA transfection. 48 hours post-transfection, cells underwent fixation in 4% paraformaldehyde for 10 minutes at room temperature, followed by washing. Cells were then permeabilized using 0.5% Triton X-100 in PBS for 10 minutes at room temperature, succeeded by an additional PBS wash. Initial incubation occurred in a saturation buffer composed of 3% BSA in TBS 1X with 0.1% Tween for 1 hour. Subsequently, cells were incubated with primary antibodies: anti-ceramide (MID 15B4, EnzoLifeSciences) or anti-53BP1 (ab21083, Abcam) and anti-γH2AX (05–636, Merck) for 1.5 hours at room temperature. Following three washes, cells were exposed to secondary antibodies, either goat-anti-mouse-IgM (SA5–10150, ThermoFisher) or goat anti-mouse AF488 and goat anti-rabbit AF594 from Molecular Probes, for 1 hour. This was followed by three additional washes and final staining with DAPI in mounting medium. Ceramide fluorescence was quantified on imageJ software by measuring the total fluorescence intensity per image after background subtraction and normalizing to the number of nuclei. Images were acquired on an AxioImager Z1 microscope using the Axio Vision software (Zeiss). DNA damage foci were quantified with the ImageJ software.

### ROS quantification

Cells were plated at 1.0 × 10^5^ per well in six-well plates 24 hours prior to siRNA transfection. ROS levels were quantified employing the CellROX Deep Red Reagent Kit (Thermofisher, C10422), according to the manufacturer’s instructions. After 48 hours of incubation, ROS levels were assessed through flow cytometry, using a CytoFLEX flow cytometer system for measurement.

### Quantitative reverse transcriptase-polymerase chain reaction (qRT-PCR)

RNA was isolated using RNeasy mini-Kit (Qiagen). Reverse transcriptase reaction (RT) was performed with 100 ng of total RNA using the Vilo mastermix superscript kit followed by quantitative polymerase chain reaction (qPCR) using Fast SYBR Green Master Mix (Thermofischer, 4385617) on a StepOne Real Time PCR System (Thermoscientific). Expression levels were normalized by *hprt*. Primer sequences are available on demand.

### Western blot

Cells were harvested from a confluent 6-well plate by scraping and subsequently washed with PBS. Cell pellets were lysed using 1X RIPA Buffer supplemented with a cocktail of phosphatase/protease inhibitors, followed by centrifugation at 4°C for 15 minutes at 1300 rpm. The supernatant was then normalized using the Bradford assay. Proteins were denatured by heating with 1X SDS-PAGE Protein Loading Buffer for 5 minutes at 85°C. Denatured proteins were loaded onto 4–15% Mini-PROTEAN TGX Precast Protein Gels (Bio-Rad) for electrophoresis. Following electrophoresis, proteins were transferred onto nitrocellulose membranes using the Trans-Blot Turbo System (Bio-Rad). Protein transfer quality was assessed with Ponceau S staining. After multiple washes in 0.1% TBS-Tween, non-specific binding sites on the membranes were blocked for 1 hour at room temperature in a solution containing 3% BSA in TBS 1X with 0.1% Tween. Membranes were then incubated overnight with primary antibodies specific to the target proteins (listed below). This was followed by three washes in 0.1% TBS-Tween before incubation with secondary antibodies for 40 minutes. After three additional washes, protein bands were visualized using Pierce ECL Western Blotting Substrate (Thermofisher).

### RNA-seq

RNA**-**seq was performed on poly(A) selected RNA extracted from 10^6^ cells of human PD20 cell line using the RNeasy Plus Mini kit (Qiagen) following manufacturer’s protocol. Poly-(A)-selected, first stranded Illumina libraries were prepared with a modified TruSeq protocol using dUTP method (*47*). Three biological replicates per conditions were prepared. Libraries were size selected using AMPure XP SPRI beads, amplified by PCR and purified with AMPure XP beads and paired-end sequenced (75 bp) on an Illumina NextSeq 500 sequencer.

Sequencing reads were pseudo aligned to the ENSEMBL GRCh38 version of the human genome and abundance quantified using Kallisto (*48*). Differential expression analysis was done using Sleuth R package (*49*). Volcano plots and heatmap were plotted using R packages ggplot2 and pheatmap.

Mature BM cells were depleted using the direct lineage cell depletion kit (Miltenyi Biotec, Paris France) following manufacturer instructions. The remaining Lin^neg^ cells were stained with Pacific Blue Mouse Lineage Antibody Cocktail, anti-CD117PercpCy5.5, ant-Sca-1 PECy7. LSK populations were purified by cell sorting using a FACS-ARIAIII (BD Bioscience). Total RNA was isolated using RNeasy Micro Kit Qiagen). For library construction mRNA was enriched from total RNA and submitted to fragmentation, cDNA synthesis, ligation of adapter and PCR amplification using the CATS RNA-seq Kit v2 x24 (Diagenode #C05010042) technology. Sequencing was performed using Nextseq500 (Illumina).

### Gene Set Enrichment Analysis

Gene set enrichment was performed using R package fgsea GSEA using MsigDB (v7.4) C2, C5 and Hallmark collections. Gene sets with less than 15 genes or with more than 500 genes were excluded from the analysis. GSEA was performed using weighted enrichment statistic on a pre-ranked list of genes after filtering out lowly expressed genes. Genes were ranked using their log_2_ fold change of expression between compared. Gene sets with an FDR ≤ 0.25 and a nominal *P* value ≤ 0.05 were considered as significant.

### Lipidomics

For lipidomic analysis, lipids from PD20 cells were extracted using a modified Bligh and Dyer protocol. Briefly samples supplemented with a mixture of internal standards were mixed with 1600 μl of acidified methanol:0.1 N HCl (1:1 v/v) and 800 μl chloroform as described elsewhere (*50*, *51*). The lower organic phase was dried; lipids were reconstituted into 40 μl of LC/MS compatible solvent and lipids quantified by LC-ESI/MS/MS using a prominence UFLC and a QTrap 4000 mass spectrometer. For quantification of phospholipid and sphingolipids, sample (4 μl) was injected to a kinetex HILIC 2.6 μm 2.1x150mm column. Mobile phases consisted of water and acetonitrile containing 30 mM ammonium acetate and 0.2% acetic acid. Lipid species were detected using scheduled multiple reaction monitoring (sMRM) in the positive-ion mode reflecting the headgroup fragmentation of each lipid class. For quantification of neutral lipids (CE, FC, DAG and TAG), sample (4 μl) was injected to an Ascentis C18 2.7 μm 2.1x150mm column. Mobile phases consisted of A [acetonitrile/water (60:40)] and B [isopropanol/acetonitrile (90:10)] in the presence of ammonium formate and formic acid. Lipid species were detected using sMRM in the positive-ion mode reflecting the neutral loss of (RCOO + NH_3_) for DAG and TAG, the product ion scan of m/z 369 (cholesterol – H2O) for CE and FC. Quantification of phospholipids, sphingolipids and neutral lipids was performed in positive-ion mode. Sample was injected to a Kinetex HILIC 2.6 μm 2.1x150mm column (Phenomenex, CA, USA). Mobile phases consisted of water and acetonitrile containing ammonium acetate and acetic acid. Lipid species were detected using scheduled multiple reaction monitoring (sMRM) and quantified using thirty-seven calibration curves specific for the 16 individual lipid subclasses and up to 12 fatty acid moieties. More abundant lipid species were quantified from a 10-fold diluted sample. An in-house developed R script was used to correct for isotopic contribution to MRM signals from HILIC separation as adapted from Ejsing CS *et al*. (*52*). All internal standards were purchased from Avanti Polar Lipids (Alabaster, AL, USA). LC/MS grade or UPLC grade solvents were used without further purification and obtained from Sigma-Aldrich (St Louis, MO, USA). Statistical analysis and heatmap clusterisation was performed with MetaboAnalyst software 5.0 (*53*).

### Quantification and statistical analysis

All statistical analyses were performed by GraphPad PRISM 9. Anova test was used for the comparisons of the means of more than two groups. *n* represents biological replicates in cell culture experiments and the number of mice in animal studies.
