# Supplementary material for: ZMAT3 alleviates cell death in Fanconi anemia via adaptation of sphingolipid metabolism
Source: Sci Adv. 2026 Jul 31;12(31):eaeb6444. doi: 10.1126/sciadv.aeb6444 (PMC13426403; doi:10.1126/sciadv.aeb6444)
Supplement: Supplementary file 1 — Figs. S1 to S4 Legends for tables S1 and S2 [file sciadv.aeb6444_sm.pdf]

Science Advances
AAAS

Supplementary Materials for

ZMAT3 alleviates cell death in Fanconi anemia via adaptation of sphingolipid metabolism

Maeva Loock *et al.*

Corresponding author: Dominique Bluteau, dominique.bluteau@ephe.psl.eu

Sci. Adv. **12**, eaeb6444 (2026)
DOI: 10.1126/sciadv.aeb6444

The PDF file includes:

Figs. S1 to S4
Legends for tables S1 and S2

Other Supplementary Material for this manuscript includes the following:

Tables S1 and S2

Fig. S1.

(A) Western blot analysis of ZMAT3 (specific band is shown by asterisk *), Vinculin (VCL), p53 and Pp53[ser15] proteins in PD20ish*ZMAT3* and control PD20ish*Ren* post 2 days with or without doxycycline (DOX) (0.5µg/mL) and with or without mitomycin (MMC) (30nM) exposure. (B) Relative ZMAT3 mRNA expression measured by qPCR and normalized to housekeeping genes (n=3). (C) Densitometric quantification of ZMAT3 protein levels from western blot analysis, normalized to loading control (n=3).

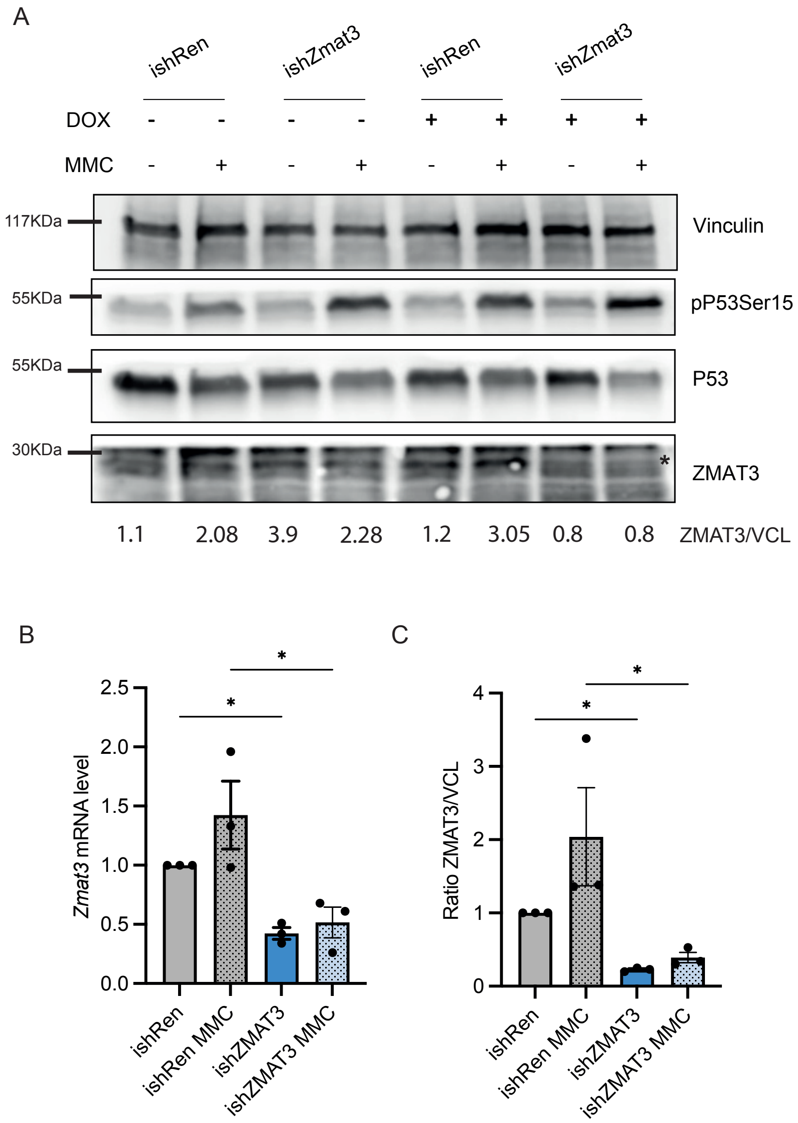

Fig. S2.

Heatmap, hierarchical clustering derived from RNA-Seq analysis, showcases significant differential gene expression between PD20ish*ZMAT3* and control PD20ishRen 2 days post DOX (0.5µg/ml) +/- MMC (30nM) exposure. Principal pathways enriched by cluster using Enrichr online software. (https://maayanlab.cloud/Enrichr/) are shown. (B) GSEA Heatmap and enrichment graphic corresponding to WP and KEGG Ferroptosis genes list. (C) *ZMAT3* and *CD44* mRNA expression measured by qPCR, normalized to housekeeping genes in PD20si*ZMAT3* relative to PD20si*ZMAT3* cells (n=5).

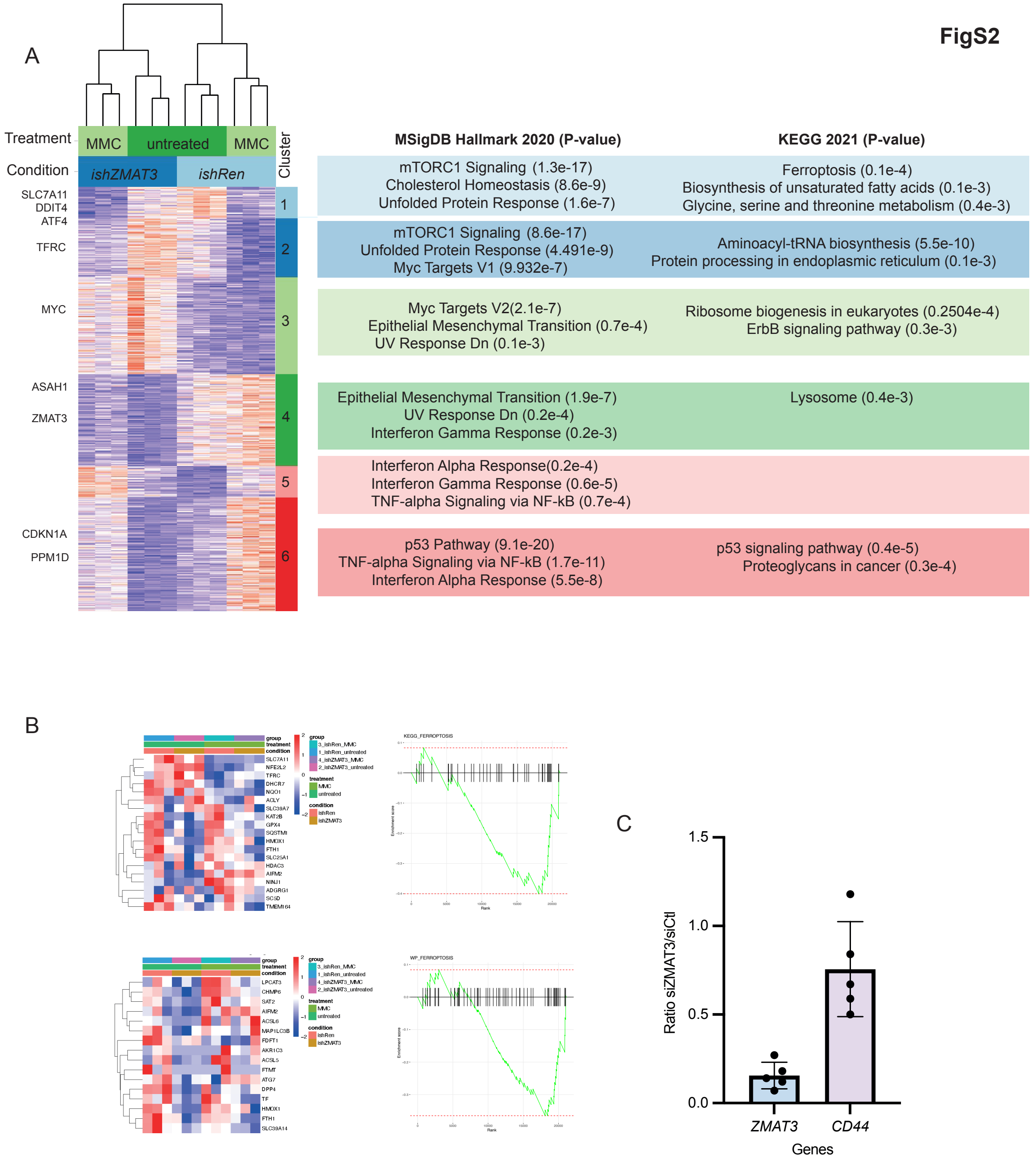

Fig. S3.

Summary and lipids pathway. Lipids in red are enriched in PD20*ishZMAT3* compared to PD20*ishRen* cells. Lipids in blue are depleted in PD20ish*ZMAT3* cells compared to PD20*ishRen* cells. In brackets, amount of lipid species significantly altered in the corresponding class l: long chain lipids (≥38 carbons) s: short chain lipids (≤36 carbons).

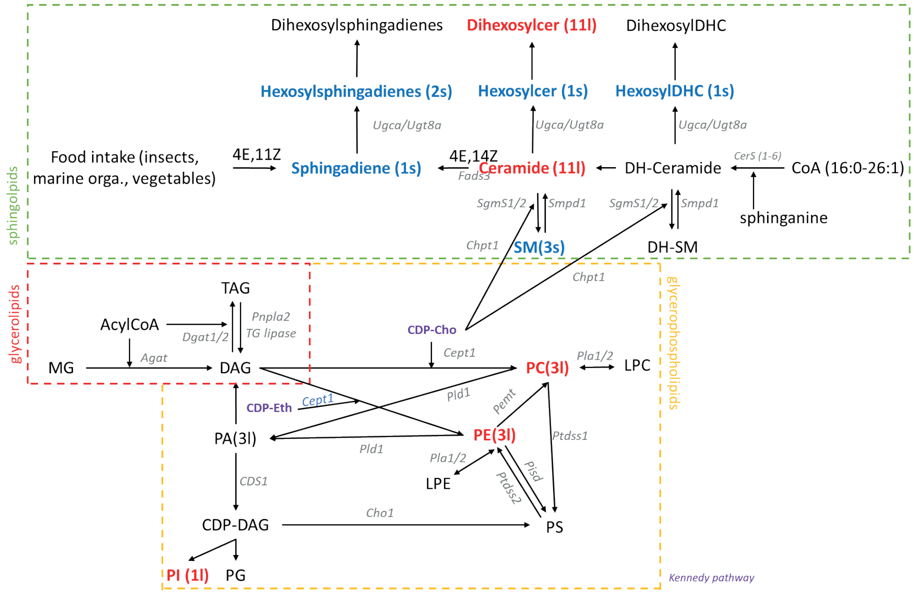

Fig. S4.

(A) Proliferation measurement in sorted PD20 cells post-72-hour siRNA transfection by quantification of the mean fluorescence intensity (MFI) of CellTrace labeling (n=4). (B) Senescence level in sorted PD20 cells post-48-hour siRNA transfection quantified through β-galactosidase hydrolysis (n=4). (C) Quantification of necrosis or (D) apoptosis level in sorted PD20 cells post-48-hour siRNA transfection, with and without a 2-hour MMC pulse (300ng/ml), followed by a 24-hour recovery period, by Sytox and Annexin V labeling respectively (n=4). Errors bars shown mean+/- SEM. p values from ordinary one ways ANOVA are shown (n=4). (E) Cell viability assay using MMT test over 2-days treatment with escalating doses of Erastin or RSL3. (n=2 with duplicate; mean+/-SEM).

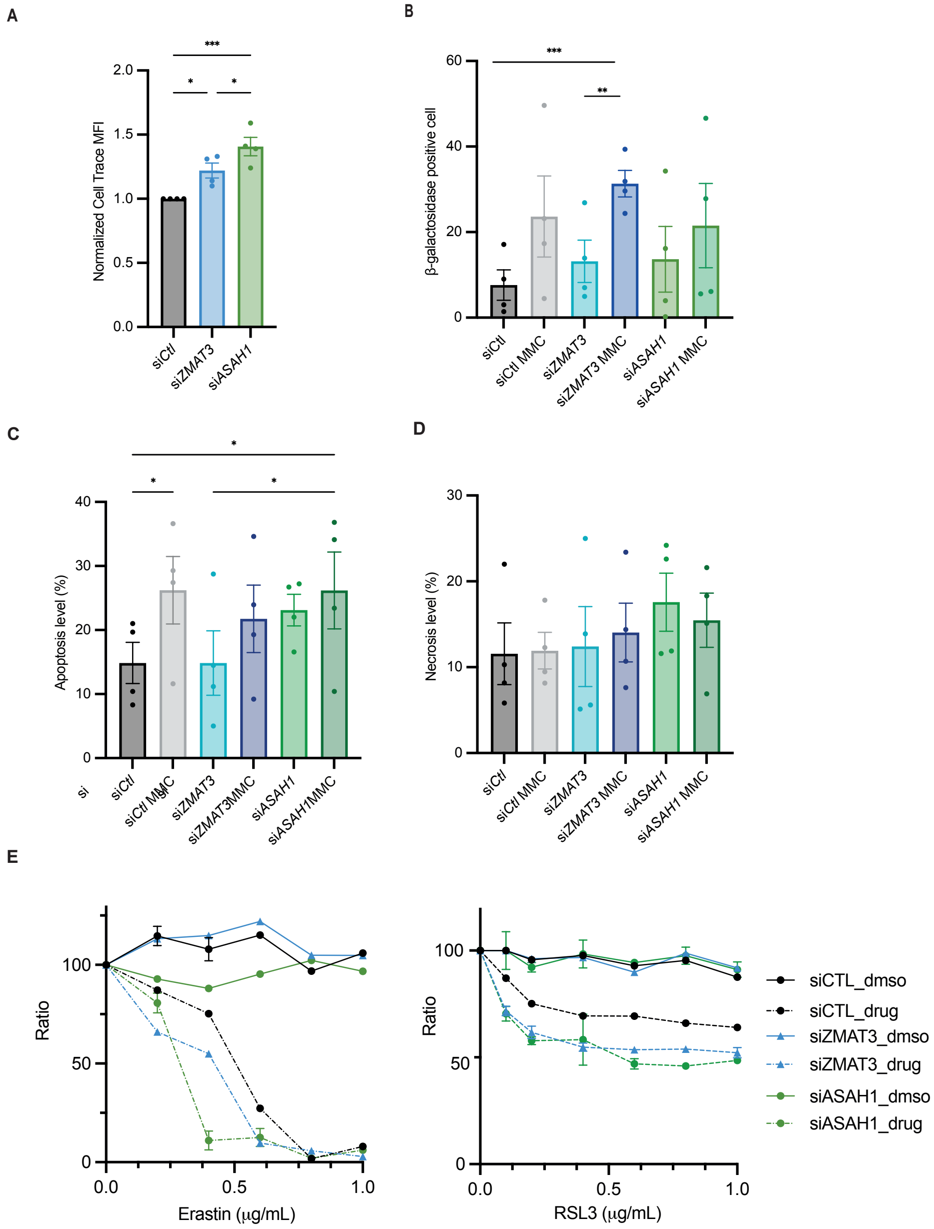

Table S1. Significant genes and clusters for comparison All vs ishRen untreated
Table S2. *Sheet 1*: lipids list (molPercent species), *Sheet 2*: lipids list (molPercent totals)
